# Effects of protein type and composition on postprandial markers of skeletal muscle anabolism, adipose tissue lipolysis, and hypothalamic gene expression

**DOI:** 10.1186/s12970-015-0076-9

**Published:** 2015-03-13

**Authors:** Christopher Brooks Mobley, Carlton D Fox, Brian S Ferguson, Corrie A Pascoe, James C Healy, Jeremy S McAdam, Christopher M Lockwood, Michael D Roberts

**Affiliations:** School of Kinesiology, Molecular and Applied Sciences Laboratory, Auburn University, 301 Wire Road, Office 286, Auburn, AL 36849 USA; 4Life Research USA, LLC, Sandy, UT USA

**Keywords:** Whey protein, Egg protein, Hydrolyzed protein, Muscle protein synthesis, Lipolysis

## Abstract

**Background:**

We examined the acute effects of different dietary protein sources (0.19 g, dissolved in 1 ml of water) on skeletal muscle, adipose tissue and hypothalamic satiety-related markers in fasted, male Wistar rats (~250 g).

**Methods:**

Oral gavage treatments included: a) whey protein concentrate (WPC, n = 15); b) 70:30 hydrolyzed whey-to-hydrolyzed egg albumin (70 W/30E, n = 15); c) 50 W/50E (n = 15); d) 30 W/70E (n = 15); and e) 1 ml of water with no protein as a fasting control (CTL, n = 14).

**Results:**

Skeletal muscle analyses revealed that compared to CTL: a) phosphorylated (p) markers of mTOR signaling [p-mTOR (Ser2481) and p-rps6 (Ser235/236)] were elevated 2–4-fold in all protein groups 90 min post-treatment (p < 0.05); b) WPC and 70 W/30E increased muscle protein synthesis (MPS) 104% and 74% 180 min post-treatment, respectively (p < 0.05); and c) 70 W/30E increased p-AMPKα (Thr172) 90 and 180-min post-treatment as well as PGC-1α mRNA 90 min post-treatment. Subcutaneous (SQ) and omental fat (OMAT) analyses revealed: a) 70 W/30 W increased SQ fat phosphorylated hormone-sensitive lipase [p-HSL (Ser563)] 3.1-fold versus CTL and a 1.9–4.4-fold change versus all other test proteins 180 min post-treatment (p < 0.05); and b) WPC, 70 W/30E and 50 W/50E increased OMAT p-HSL 3.8–6.5-fold 180 min post-treatment versus CTL (p < 0.05). 70 W/30E and 30 W/70E increased hypothalamic POMC mRNA 90 min post-treatment versus CTL rats suggesting a satiety-related response may have occurred in the former groups. However, there was a compensatory increase in orexigenic AGRP mRNA in the 70 W/30E group 90 min post-treatment versus CTL rats, and there was a compensatory increase in orexigenic NPY mRNA in the 30 W/70E group 90 min post-treatment versus CTL rats.

**Conclusions:**

Higher amounts of whey versus egg protein stimulate the greatest post-treatment anabolic skeletal muscle response, though test proteins with higher amounts of WPH more favorably affected post-treatment markers related to adipose tissue lipolysis.

**Electronic supplementary material:**

The online version of this article (doi:10.1186/s12970-015-0076-9) contains supplementary material, which is available to authorized users.

## Background

Dietary whey protein has numerous well-known health benefits. For instance, whey protein feeding has been shown to acutely increase postprandial muscle protein synthesis (MPS) in rodents [1,2] and humans [3,4], whereas chronic whey protein supplementation has been shown to consistently increase muscle mass with exercise training [5-7]. Acute whey protein feeding has also been shown to reduce appetite 90–180 min following low-dose ingestion [8-10] by potentially affecting anorectic hormone and hypothalamic mRNA expression patterns [11,8,9]. Chronic whey protein supplementation has also been shown to reduce adiposity in rodents and humans [12-14,5]; an effect which may be explained by an increased expression of adipose tissue lipolysis-related gene expression patterns following chronic supplementation [12], an increase in protein-induced thermogenesis (reviewed in [15]), and/or a consistent reduction in food intake given its satiety-stimulatory effects as discussed above.

More recent data has focused on the potential health benefits of hydrolyzed dietary proteins. In short, commercial hydrolysis of different dietary protein sources is thought to [16-18]: a) expedite the digestion of amino acids via ‘pre-digestion’ thus increasing their postprandial bioavailability; and b) liberate bioactive peptides that are able to exhibit physiological responses that otherwise would be diminished from consuming intact protein sources. Indeed, *in vivo* [19,20] and *in vitro* [21] evidence suggests that hydrolyzed whey or native whey protein increases the activation of postprandial intramuscular insulin signaling markers. Putative bioactive peptides from whey protein hydrolysates (WPH) have also been shown to exhibit insulin secretagogue properties versus intact whey protein [22,23]. Likewise, we have recently demonstrated that acute WPH feeding in rats increases the appearance of di- and oligopeptides as well as numerous lipolysis-related serum markers (i.e., epinephrine, glycerol and numerous free fatty acids) compared to an isonitrogenous WPC feeding condition [18]. Thus, it is of interest to further examine how WPH versus WPC ingestion differentially affects various physiological systems.

Widespread interest has also surrounded the positive health benefits of dietary egg protein due to its high essential amino acid (EAA) content and high digestibility [24]. Similar to whey protein, egg protein feeding in rats has been found to significantly increase postprandial MPS [1]. Likewise, one report suggests that bioactives isolated from egg protein down-regulate serum myostatin (MSTN) [25]; an effect which may enhance skeletal muscle hypertrophy with chronic supplementation. However, unlike the aforementioned whey protein research, there is a paucity of data regarding the physiological effects of dietary egg protein on other tissues (i.e., adipose tissue and the hypothalamus), though there is some evidence to suggest that egg-based breakfast meals can increase satiety post-ingestion [26] and cause weight loss in overweight individuals over the long-term [27].

Given the widespread interest regarding the physiological effects of dietary whey and egg proteins, as well as hydrolyzed versus intact protein forms, the purpose of this study was to examine how different solutions of extensively hydrolyzed whey and egg albumin protein (EPH) blends, in combination with a standardized blend of cow colostrum and egg yolk extract acutely affect post-prandial markers of skeletal muscle anabolism, adipose tissue lipolysis and thermogenesis, and hypothalamic mRNA expression patterns in rodents. Treatments included: 300 human equivalent mg of bovine colostrum and egg yolk extract (0.0057 g protein rat dose) in addition to 10 human equivalent g protein dose (0.19 g protein rat dose) of, a) high-dose WPH + low-dose EPH (70 W/30E); b) equal doses of both WPH and EPH (50 W/50E); and c) low-dose WPH + high-dose EPH (30 W/70E). An isonitrogenous amount of intact whey protein concentrate (WPC) was also fed to a fourth group of rats as a positive feeding control, and 1 ml of water with no protein was fed to a fifth group of rats as a fasting control (CTL). Based upon the aforementioned literature, we hypothesized that all protein treatments would similarly increase postprandial markers of skeletal muscle anabolism as well as satiety-related hypothalamic markers relative to CTL. We also hypothesized that higher proportions of whey protein (i.e., WPC and 70 W/30E) would induce larger increases in adipose tissue lipolysis markers relative to other feeding groups; though we also hypothesized that the hydrolysates would outperform the WPC on markers of muscle anabolism, adipose tissue lipolysis and satiety.

## Experimental methods

### Animals and feeding protocols

All experimental procedures described herein were approved by Auburn University’s Institutional Animal Care and Use Committee. Male Wistar rats (~250 g) approximately 8–9 weeks old were purchased from Harlan Laboratories and were allowed to acclimate in the animal quarters for 5 days prior to experimentation. Briefly, animal quarters were maintained on a 12 h light: 12 h dark cycle, at ambient room temperature, with water and standard rodent chow (18.6% protein, 44.2% carbohydrate, 6.2% fat; Teklad Global #2018 Diet, Harlan Laboratories) provided to animals *ad libitum*.

The day prior to acute protein feeding experiments, food was removed from home cages resulting in an 18 h overnight fast. The morning of experimentation, animals were removed from their quarters between 0800–0900, transported to the Molecular and Applied Sciences Laboratory and were allowed to acclimate for approximately 3–5 h. Thereafter, rats were administered either WPC, 70 W/30E, 50 W/50E, 30 W/70E at a human equivalent (eq.) dose of 10 g protein (0.19 g protein rat dose) dissolved in 1 ml of tap water via gavage feeding. Doses were calculated per the species conversion calculations of Reagan-Shaw et al. [28], whereby the human body mass for an average male was assumed to be 80 kg. The group of non-fed CTL rats was gavage-fed 1 ml of tap water. Dietary components of each test protein solution are presented in Table 1.

**Table 1:** **Contents of each protein per the 0.19 g protein dose of each respective protein**

**Amino Acid**	**70 W/30E (mg)**	**50 W/50E (mg)**	**30 W/70E (mg)**	**WPC (mg)**
Alanine	10	10	10	9
Arginine	8	8	9	6
Aspartic Acid	21	20	19	22
Cysteine	5	5	5	4
Glutamic Acid	31	29	26	34
Glycine	4	5	5	4
Histidine*	4	4	4	4
Isoleucine*†	11	11	10	12
Leucine*†	20	19	17	23
Lysine*	17	15	14	19
Methionine*	5	5	6	4
Phenylalanine*	8	9	9	7
Proline	12	11	9	17
Serine	12	12	11	12
Threonine*	11	10	9	12
Tryptophan*	3	3	3	3
Tyrosine	7	7	7	6
Valine*†	12	12	12	11
Total EAAs*	***92***	***87***	***83***	***95***
Total BCAAs*†	***43***	***41***	***38***	***46***
**M.W.**	**70 W/30E**	**50 W/50E**	**30 W/70E**	**WPC**
**(kDa)**	**(%)**	**(%)**	**(%)**	**(%)**
<1.0	40	39	40	0
1.0 - 5.0	23	22	23	7
5.0 - 10.0	6	6	6	11
>10.0	30	32	32	82

As stated in the methods, rats were administered either WPC, 70 W/30E, 50 W/50E, or 30 W/70E at a human equivalent (eq.) dose of 10 g protein (equaled a true dose of 0.191 g of protein) dissolved in 1 ml of water via gavage feeding. While not stated in the methods, total protein was determined using the Dumas (N x 6.38) test method. Furthermore, amino acid concentrations (g/100 g) were determined using liquid chromatography. Finally, molecular weight (M.W.) distribution was determined by high-performance liquid chromatography size exclusion (HPLC-SEC), on an Agilent 1290 Infinity Quaternary LC System w/ TOSOH TSKgel G2000SW 7.5 mm ID × 30 cm (10 μm) column at a wavelength of 205 nm. Symbols: * indicates an essential amino acid; † indicates a branched chain amino acid (BCAA).

Of note, We examined how graded doses of WPC in solution (0.19, 0.37, and 0.93 g protein) stimulated MPS and Akt-mTOR markers 90 min post-gavage in order to determine an optimal dose that adequately elicited a post-prandial physiological response. These preliminary results demonstrated that 10 human eq. g of WPC (0.19 g protein) increased markers of mTOR activation and MPS 90 min post-gavage, and this generally was equal to the 19 human eq. g (0.37 g protein) and 48 human eq. g (0.93 g protein) doses (Additional file 1: Figure S1). Thus, given that the 10 human eq. g of WPC (0.19 g protein) elicited similar anabolic responses compared to higher doses, we opted to use the 10 human eq. g (0.19 g) dose for each test protein. While this dose is not typically associated with the optimal human MPS response to protein ingestion (i.e., 20–40 g), it should be noted that the species conversion calculations of Reagan-Shaw et al. is a basis to dose rats relative to humans and, alternatively, these human eq. dosages should not be viewed in absolute terms when comparing species (i.e., 10 human eq. g appears to elicit an anabolic response in rats whereas 20–40 g in humans is needed).

The gavage feeding procedure involved placing the animals under light isoflurane anesthesia for approximately 1 min while gavage feeding occurred. Following gavage feeding, rats were allowed to recover 90 or 180 min prior to being euthanized under CO_2_ gas in a 2 L induction chamber (VetEquip, Inc., Pleasanton, CA, USA). Animals that were sacrificed 180 min post-treatment were injected intraperitoneally with puromycin dihydrochloride (5.44 mg in 1 ml of diluted in phosphate buffered saline; Ameresco, Solon, OH, USA) 30 min prior to euthanasia in order to determine skeletal muscle protein synthesis via the surface sensing of translation (SUnSET) method described in detail elsewhere [29]. Of note, with the SUnSET method MPS is determined through the incorporation of puromycin into actively synthesized proteins given that it is a structural analogue of aminoacyl-transfer RNA; specifically tyrosyl-tRNA. It should also be noted that the SUnSET method is an alternative method for measuring MPS compared to radioactive isotope (e.g. ^3^H-phenyalanine or ^35^S-methionine), or stable isotope (e.g.^15^ N-lysine, ^13^C-leucine or [ring-^13^C6]-phenylalanine) tracers. Goodman et al. [29] compared the SUnSET method to a ^3^H-phenyalanine flooding method in *ex vivo* plantaris muscle preparations isolated from animals that had undergone synergist ablation. Remarkably, these authors determined that MPS rates increased 3.6-fold as determined by the SUnSET method and 3.4-fold as determined by the tracer method; a finding which proves the reliability of this method in detecting sensitive changes in MPS.

Immediately following euthanasia, whole blood was removed via heart sticks using a 21-gauge needle and syringe, placed in a serum separator tubes, and processed for serum extraction via centrifugation at 3,500 × *g* for 5 min. Serum was aliquoted into multiple 1.7 ml microcentrifuge tubes for subsequent biochemical assays and then frozen for later analysis. Approximately two 50 mg pieces of mixed gastrocnemius muscle was harvested using standard dissection techniques and placed in homogenizing buffer [Tris base; pH 8.0, NaCl, NP-40, sodium deoxycholate, SDS with added protease and phosphatase inhibitors (G Biosciences, St. Louis, MO, USA)] and Ribozol (Ameresco) for immunoblotting and mRNA analyses, respectively. Approximately two 50 mg pieces of subcutaneous adipose tissue (SQ) from the inguinal crease was harvested and placed in the aforementioned Tris base homogenizing buffer and Ribozol for immunoblotting and mRNA analyses, respectively. Due to tissue limitations, only one 50 mg piece of omental adipose tissue (OMAT) was harvested and placed in the aforementioned Tris base homogenizing buffer for immunoblotting. Finally, removal of the hypothalamus was performed per the methods similar to those previously employed [30]. Briefly, brains were removed and rinsed in 1x phosphate buffered saline. Brains were then placed posterior side up in a 1.0 mm acrylic sectioning apparatus (Braintree Scientific, Braintree, MA, USA) and a 2.0-mm coronal slice of each brain was made between Bregma-1.6 and-1.8 mm. Coronal slices were immediately placed on an ice-cooled stage and two bilateral punches (2.0 mm diameter) were made to capture the hypothalamus. Tissue was immediately placed in Ribozol and stored at-80°C until RNA isolation.

Gastrocnemius muscle, SQ and OMAT samples placed in Tris base homogenizing buffer were homogenized using a 1.7 ml tube using a tight-fitting micropestle, insoluble proteins were removed with centrifugation at 500 × *g* for 5 min at 4°C, and supernatants were assayed for total protein content using a BCA Protein Assay Kit (Thermo Scientific, Waltham, MA, USA) prior to immunoblotting sample preparation. Muscle, SQ, and hypothalamus samples placed in Ribozol were subjected to total RNA isolation according to manufacturer’s instructions, and concentrations were performed using a NanoDrop Lite (Thermo Scientific) prior to cDNA synthesis for mRNA analyses. Extra gastrocnemius muscle and SQ fat not processed during dissections were flash-frozen in liquid nitrogen and stored at-80°C for later potential analyses.

### Directed Akt-mTOR phosphoproteomics

The PathScan® Akt Signaling Antibody Array Kit (Chemiluminescent Readout; Cell Signaling, Danvers, MA, USA) containing glass slides spotted with antibodies was utilized to detect phosphorylated proteins predominantly belonging to the Akt-mTOR signaling network.

The kit assays p-Akt (Thr308), p-Akt (Ser473), p-rps6 (Ser235/236), p-AMPKα (Thr172), p-Pras40 (Thr246), p-mTOR (Ser2481), p-GSK-3α (Ser21), p-GSK-3β (Ser9), p-p70s6k (Thr389), p-p70s6k (Thr421/Ser424), p-BAD (Ser112), p-PTEN (Ser380), p-PDK1 (Ser241), p-ERK1/2 (Thr202/Tyr204), p-4E-BP1 (Thr37/46). However, we specifically analyzed p-Akt (Ser473), p-rps6 (Ser235/236), p-AMPKα (Thr172), p-mTOR (Ser2481), p- p-p70s6k (Thr389), and p-4E-BP1 (Thr37/46) in order follow a ‘linear’ analysis in Akt-mTOR signaling. Briefly, gastrocnemius homogenates were diluted to 0.5 μg/μl using cell lysis buffer provided by the kit and assayed according to manufacturer’s instructions. Slides were developed using an enhanced chemiluminescent reagent provided by the kit, and spot densitometry was performed through the use of a UVP Imager and associated densitometry software (UVP, LLC, Upland, CA, USA). The calculation of each phosphorylated target was as follows:

(Density value of the target – negative control)/summation of all density values for the sample.

It should be noted that this high throughput antibody chip array for muscle phosphorylation markers was used rather than single antibodies due to resource constraints. Notwithstanding, and as discussed in the results section, the results presented herein are in agreement with past literature showing that protein feeding affects numerous targets on the aforementioned antibody array chip. Furthermore, our preliminary WPC graded-dose feedings show an increase in Akt-mTOR markers across multiple doses relative to fasting rats (Additional file 1: Figure S1). We have also internally tested this array on exercised rat muscle as well as C2C12 cell culture lysates deprived of or treated with L-leucine, and have produced reproducible results commensurate with prior literature examining these markers (i.e., increased activation of mTOR markers which parallel increases in MPS; *unpublished observations*).

### Western blotting

As mentioned prior, the SUnSET method was employed in order to examine if different dietary protein blends differentially affected MPS. Briefly, 2 μg/μl gastrocnemius Western blotting preps were made using 4x Laemmli buffer. Thereafter, 20 μl of prepped samples were loaded onto pre-casted 4–20% SDS-polyacrylamide gels (C.B.S. Scientific Company, San Diego, CA, USA) and subjected to electrophoresis (200 V @ 75 min) using pre-made 1x SDS-PAGE running buffer (C.B.S. Scientific Company). Proteins were then transferred to polyvinylidene difluoride membranes, and membranes were blocked for 1 h at room temperature with 5% nonfat milk powder. For muscle samples, mouse anti-puromycin IgG (1:5,000; Millipore) was incubated with membranes overnight at 4°C in 5% bovine serum albumin (BSA), and the following day membranes were incubated with anti-mouse IgG secondary antibodies (1:2,000, Cell Signaling) at room temperature for 1 h prior to membrane development described below. Thereafter, membranes were stripped of antibodies via commercial stripping buffer (Restore Western Blot Stripping Buffer, Thermo Scientific), membranes were incubated with rabbit anti-beta-actin (1:5,000; GeneTex, Inc., Irvine, CA, USA) as a normalizer protein overnight at 4°C in 5% BSA, and the following day membranes were incubated with anti-rabbit IgG secondary antibodies (1:2,000, Cell Signaling) at room temperature for 1 h prior to membrane development.

SQ and OMAT samples were assayed with rabbit anti-phospho-hormone sensitive lipase [p-HSL (Ser563) IgG (1:1000; Cell Signaling)] overnight at 4°C in 5% BSA. The following day membranes were incubated with anti-rabbit IgG secondary antibodies (1:2,000, Cell Signaling) at room temperature for 1 h prior to membrane development. Membranes were stripped, incubated with rabbit glyceraldehyde 3-phosphate dehydrogenase (GAPDH; 1:5,000; GeneTex) overnight at 4°C in 5% BSA, and the following day were incubated with anti-rabbit IgG secondary antibodies (1:2,000, Cell Signaling) at room temperature for 1 h prior to membrane development.

Membrane development was performed using an enhanced chemiluminescent reagent (Amersham, Pittsburgh, PA, USA), and band densitometry was performed through the use of a UVP Imager and associated densitometry software (UVP, LLC, Upland, CA, USA).

### Real-time RT-PCR

RNA from each tissue (500 ng of hypothalamus RNA and 1 μg of gastrocnemius and SQ RNA) were reverse transcribed into cDNA for real time PCR analyses using a commercial cDNA synthesis kit (Quanta Biosciences, Gaithersburg, MD, USA). Real-time PCR was performed using SYBR-green-based methods with gene-specific primers [MSTN, Mighty/Akirin-1, Myosin Heavy Chain 4 (Myhc4), p21Cip1, Atrogin-1, MuRF-1, GLUT-4, Insulin-like growth factor-1ea (IGF-1Ea), proopiomelanocortin (POMC), neuropeptide Y (NPY), agouti-related protein (AGRP), leptin receptor (LEPR), peroxisome proliferator-activated receptor gamma co-activator 1-alpha (PGC-1α), uncoupling protein 3 (UCP3), carnitine palmitoyltransferase 1b (CPT1B), beta-2 microglobulin (B2M), and beta-actin] designed using primer designer software (Primer3Plus, Cambridge, MA, USA). The forward and reverse primer sequences are as follows: [MSTN: forward primer 5′-ACGCTACCACGGAAACAATC-3′, reverse primer 5′-CCGTCTTTCATGGGTTTGAT-3′; Mighty/Akirin-1: forward primer 5′-TTTGATCTTGGGGATTCTGG-3′, reverse primer 5′-GCCTGGAAACAGTCCCTGTA-3′; p21Cip1: forward primer 5′-AGCAAAGTATGCCGTCGTCT-3′, reverse primer 5′-ACACGCTCCCAGACGTAGTT-3′; Atrogin-1: forward primer 5′-CTACGATGTTGCAGCCAAGA −3′, reverse primer 5′- GGCAGTCGAGAAGTCCAGTC-3′; MuRF-1: forward primer 5′-AGTCGCAGTTTCGAAGCAAT-3′, reverse primer 5′-AACGACCTCCAGACATGGAC-3′; GLUT-4: forward primer 5′-GCTTCTGTTGCCCTTCTGTC-3′, reverse primer 5′-TGGACGCTCTCTTTCCAACT-3′; IGF-1Ea: forward primer 5′-TGGTGGACGCTCTTCAGTTC-3′, reverse primer 5′-TCCGGAAGCAACACTCATCC-3′; POMC: forward primer 5′-GAAGGTGTACCCCAATGTCG-3′, reverse primer 5′-CTTCTCGGAGGTCATGAAGC-3′; NPY: forward primer 5′-AGAGATCCAGCCCTGAGACA-3′, reverse primer 5′-AACGACAACAAGGGAAATGG-3′; AGRP: forward primer 5′-CGTGTGGGCCCTTTATTAGA-3′, reverse primer 5′-CAGACCTTCTGATGCCCTTC-3′; LEPR: forward primer 5′-CTGGGTTTGCGTATGGAAGT-3′, reverse primer 5′-CCAGTCTCTTGCTCCTCACC-3′; PGC-1α: forward primer 5′-ATGTGTCGCCTTCTTGCTCT-3′, reverse primer 5′-ATCTACTGCCTGGGGACCTT-3′; UCP3: forward primer 5′-GAGTCAGGGGACTGTGGAAA-3′, reverse primer 5′-GCGTTCATGTATCGGGTCTT-3′; CPT1B: forward primer 5′-CCCAGTTCTGAGACCAGCTC-3′, reverse primer 5′-TAGGCACCTAAGGGCTGAGA-3′; B2M: forward primer 5′-CCCAAAGAGACAGTGGGTGT-3′, reverse primer 5′-CCCTACTCCCCTCAGTTTCC-3′; beta-actin: forward primer 5′-GTGGATCAGCAAGCAGGAGT-3′, reverse primer 5′-ACGCAGCTCAGTAACAGTCC-3′] and SYBR green chemistry (Quanta). Primer efficiency curves for all genes were generated and efficiencies ranged between 90% and 110%, and melt curve analyses demonstrated that one PCR product was amplified per reaction.

### SQ cAMP determination

Frozen SQ samples were subjected to 3'–5'-cyclic adenosine monophosphate (cAMP) assays using a rat-specific spectrophotometric commercial assay (R&D Systems, Inc., Minneapolis, MN, USA). Briefly, approximately 50–100 mg of tissue was homogenized in 500 μl of 0.1 N HCl. Samples were subjected to 10 min of centrifugation at 10,000 × g at 4°C, and neutralized with 50 μl of 1 N NaOH. Samples were then diluted 2-fold with the assay diluent provided, and cAMP concentrations were determined according to the manufacturer’s recommendations.

### Serum analyses

Serum samples were assayed for lipolysis markers including free fatty acids (FFAs) as well as epinephrine (EPI) and norepinephrine (NorEPI) using rat-specific spectrophotometric commercial assays according to the manufacturer’s recommendations (FFAs: Abcam, Cambridge, MA, USA; EPI/NorEPI: Abnova, Taipei City, Taiwan). Serum samples were also analyzed for triiodothyronine (T3) using a rat-specific spectrophotometric commercial assay according to the manufacturer’s recommendations (Abnova).

### Statistics

All data are presented in figures and tables as means ± standard error values. Given that each post-treatment time point were comprised of independent groups of rats, statistical comparisons were performed using one-way ANOVAs, and statistical significance was set at p < 0.05 (SPSS v 22.0, IBM, Armonk, NY, USA). When between-group significance was obtained, a Fisher’s LSD *post hoc* test was performed in order to determine specific between-group comparisons.

## Results

### A higher proportion of whey protein versus egg protein elicits the most favorable postprandial anabolic response

**Figure 1:**
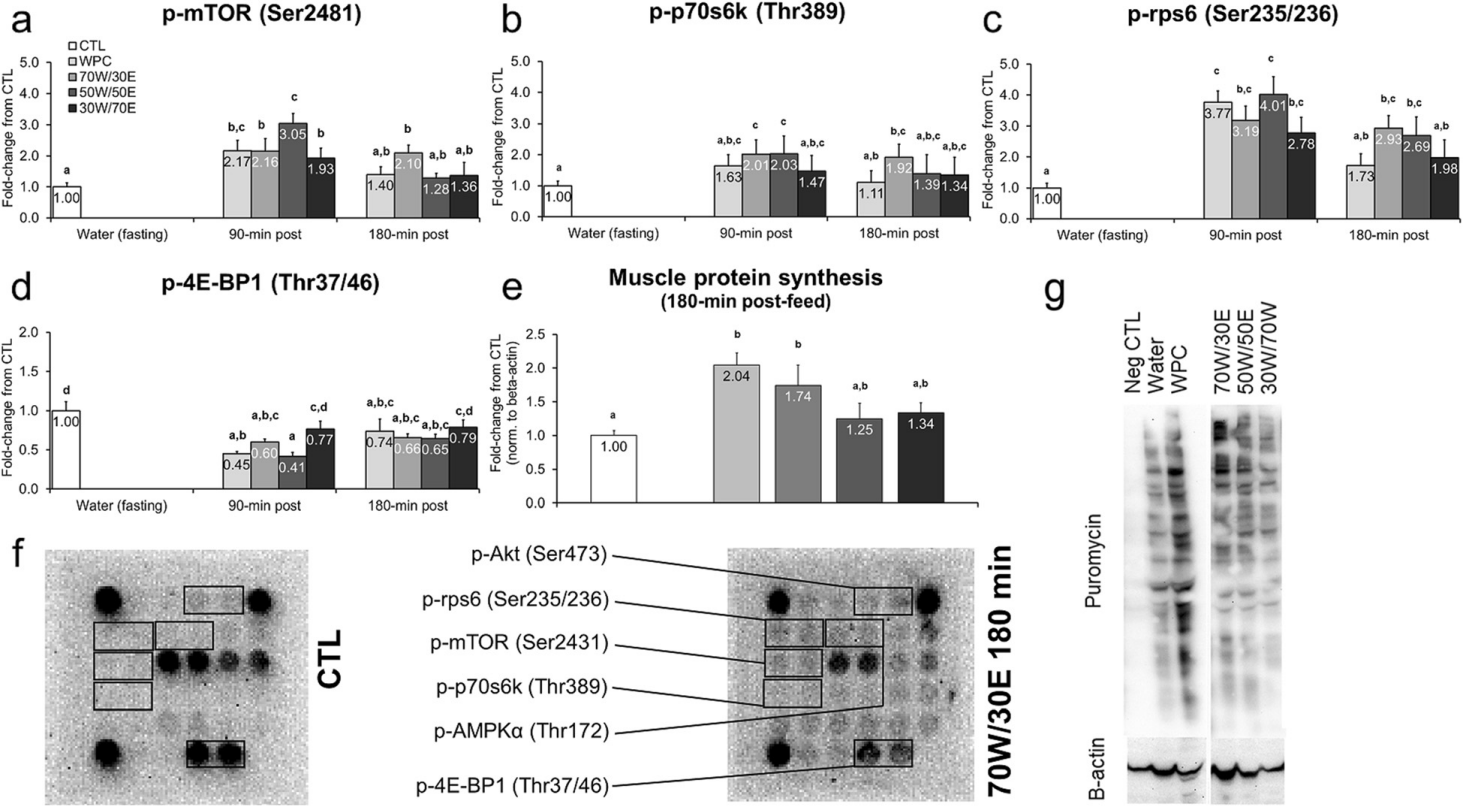
**Effects of different protein feedings on skeletal muscle anabolism markers.** Legend: Effects of each protein on gastrocnemius p-mTOR (Ser2481) (**panel a**), p-p70s6k (Thr389) (**panel b**), p-rps6 (Ser235/236) (**panel c**), p-4E-BP1 (Thr37/46) (**panel d**), and muscle protein synthesis (MPS) (**panel e**). Data are presented as means ± standard error (CTL n = 12–14 per bar, protein groups n = 6–8 per bar). One-way ANOVAs with a Fisher’s LSD *post hoc* test were performed and significant between-feeding differences are represented with different superscript letters (p < 0.05). **Panel f**: Example digital images of Akt-mTOR substrates of CTL rats and 70 W/30E-fed rats 180 min post-gavage. **Panel g**: Representative digital images of puromycin integration into muscle protein (SUnSET determination of MPS).

mTOR pathway targets were assayed in order to determine how each protein source affect post-prandial Akt-mTOR signaling substrates which, when activated, lead to increases in MPS. p-mTOR (Ser2481) was approximately 2-to-3-fold greater for protein-fed versus CTL rats 90 min post-gavage (WPC vs. CTL p = 0.006, 70 W/30E vs. CTL p = 0.005, 50 W/50E vs. CTL p < 0.001, 30 W/70E p = 0.022; Figure 1a), though it only remained significantly elevated in the 70 W/30E group 180 min post-gavage compared to CTL rats (p = 0.010; Figure 1a). p-p70s6k (Thr389) was significantly elevated approximately 2-fold in 70 W/30E and 50 W/50E versus CTL rats 90 min post-feeding (70 W/30E vs. CTL p = 0.011, 50 W/50E vs. CTL p = 0.007; Figure 1b), and this marker remained significantly elevated in 70 W/30E versus CTL rats 180 min post-feeding (~1.9-fold, p = 0.020; Figure 1b). p-rps6 (Ser235/236) was approximately 2.8-to-4-fold greater for protein-fed versus CTL rats 90 min post-gavage (WPC vs. CTL p < 0.001, 70 W/30E vs. CTL p < 0.001, 50 W/50E vs. CTL p < 0.001, 30 W/70E p = 0.003; Figure 1c), and this marker remained 2.7-to-2.9-fold elevated 70 W/30E and 50 W/50E versus CTL rats 180 min post-feeding (70 W/30E vs. CTL p = 0.002, 50 W/50E vs. CTL p = 0.007; Figure 1c). Interestingly, except for the 30 W/70E group, all protein-fed groups presented statistically 30–50% lower p-4E-BP1 (Thr37/47) values 90 min (WPC vs. CTL p < 0.001, 70 W/30E vs. CTL p = 0.003, 50 W/50E vs. CTL p < 0.001, 30 W/70E p = 0.064; Figure 1d) and 180 min (WPC vs. CTL p = 0.036, 70 W/30E vs. CTL p = 0.009, 50 W/50E vs. CTL p < 0.011, 30 W/70E p = 0.107; Figure 1d) post-feeding versus CTL rats. MPS levels were higher in WPC and 70 W/30E versus CTL rats 180 min post-feeding (WPC vs. CTL p = 0.007, 70 W/30E vs. CTL p = 0.032; Figure 1e), though there was no statistical differences between protein feeding groups.

### Select gastrocnemius mRNAs related to skeletal muscle hypertrophy are differentially affected by protein type

**Figure 2:**
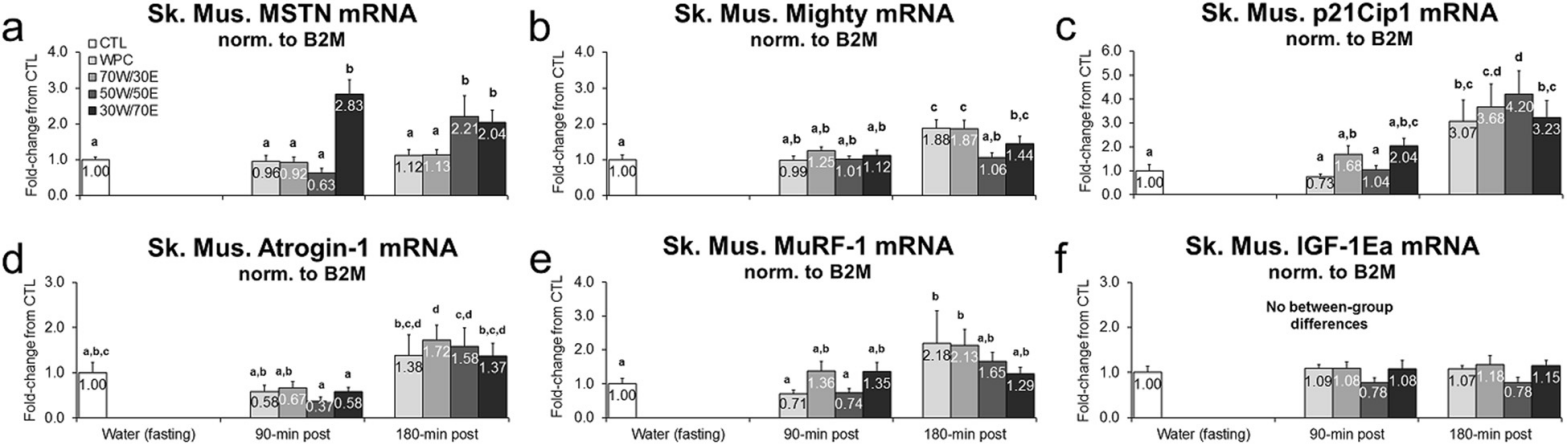
**Effects of different proteins on post-treatment gastrocnemius hypertrophy-related mRNA expression patterns.** Legend: Effects of each protein on gastrocnemius MSTN mRNA (**panel a**), Akirin-1/Mighty mRNA (**panel b**), p21Cip1 mRNA (**panel c**), Atrogin-1 mRNA (**panel d**), MuRF-1 mRNA (**panel e**), and IGF-1Ea mRNA (**panel f**). Data are presented as means ± standard error (CTL n = 12–14 per bar, protein groups n = 6–8 per bar). One-way ANOVAs with LSD *post hoc* test were performed and significant between-feeding differences are represented with different superscript letters (p < 0.05).

While transient gene expression patterns in response to feeding provide limited information, mRNA expression patterns of anabolic genes are a putative index regarding whether or not a particular protein source may have a potential impact on long-term anabolism. MSTN mRNA increased in the 30 W/70E group versus fasting rats 90 min post-feeding (p < 0.001; Figure 2a), and 30 W/70E and 50 W/50E increased MSTN mRNA 180 min post-feeding versus CTL rats (30 W/70E p = 0.003, 50 W/50E p < 0.001; Figure 2a). Mighty/Akirin-1 mRNA, which is transcriptionally down-regulated by MSTN [31] and is related to muscle hypertrophy [32], was similar between groups 90 min post-treatment but: a) was greater in the WPC and 70 W/30E groups 180 min post-treatment compared to 50 W/50E rats (WPC vs. 50 W/50E p = 0.001, 70 W/30E vs. 50 W/50E p = 0.001; Figure 2b); and b) was greater in the WPC, 70 W/30E and 30 W/70E groups 180 min post-treatment compared to CTL rats (WPC vs. CTL p < 0.001, 70 W/30E vs. CTL p < 0.001, 30 W/70E vs. CTL p = 0.046; Figure 2b). p21Cip mRNA, which is a gene potentially related to skeletal muscle hypertrophy [33], remained similar between CTL and all protein-fed groups 90 min post-feeding (Figure 2c). However, p21Cip mRNA generally increased 3–4-fold in all protein groups 180 min post-treatment versus CTL rats and 90 min post-treatment values (WPC vs. CTL p = 0.005, 70 W/30E vs. CTL p = 0.001, 50 W/50E p < 0.001, 30 W/70E vs. CTL p = 0.004; Figure 2c). Atrogin-1 mRNA remained unaltered 90 min post-feeding in all protein groups compared to CTL rats, but increased in the 70 W/30E 180 min post-feeding versus CTL rats (p = 0.049; Figure 2d). MuRF-1 mRNA remained unaltered 90 min post-feeding in all protein groups compared to CTL rats, but was greater 180 min post-feeding in the WPC and 70 W/30E groups versus CTL rats at this time point (WPC vs. CTL p = 0.020, 70 W/30E vs. CTL p = 0.032; Figure 2e). No between-group differences existed for IGF-1Ea expression patterns (Figure 2f).

### Select gastrocnemius metabolic-related phosphoprotein and mRNAs are differentially affected by protein type

**Figure 3:**
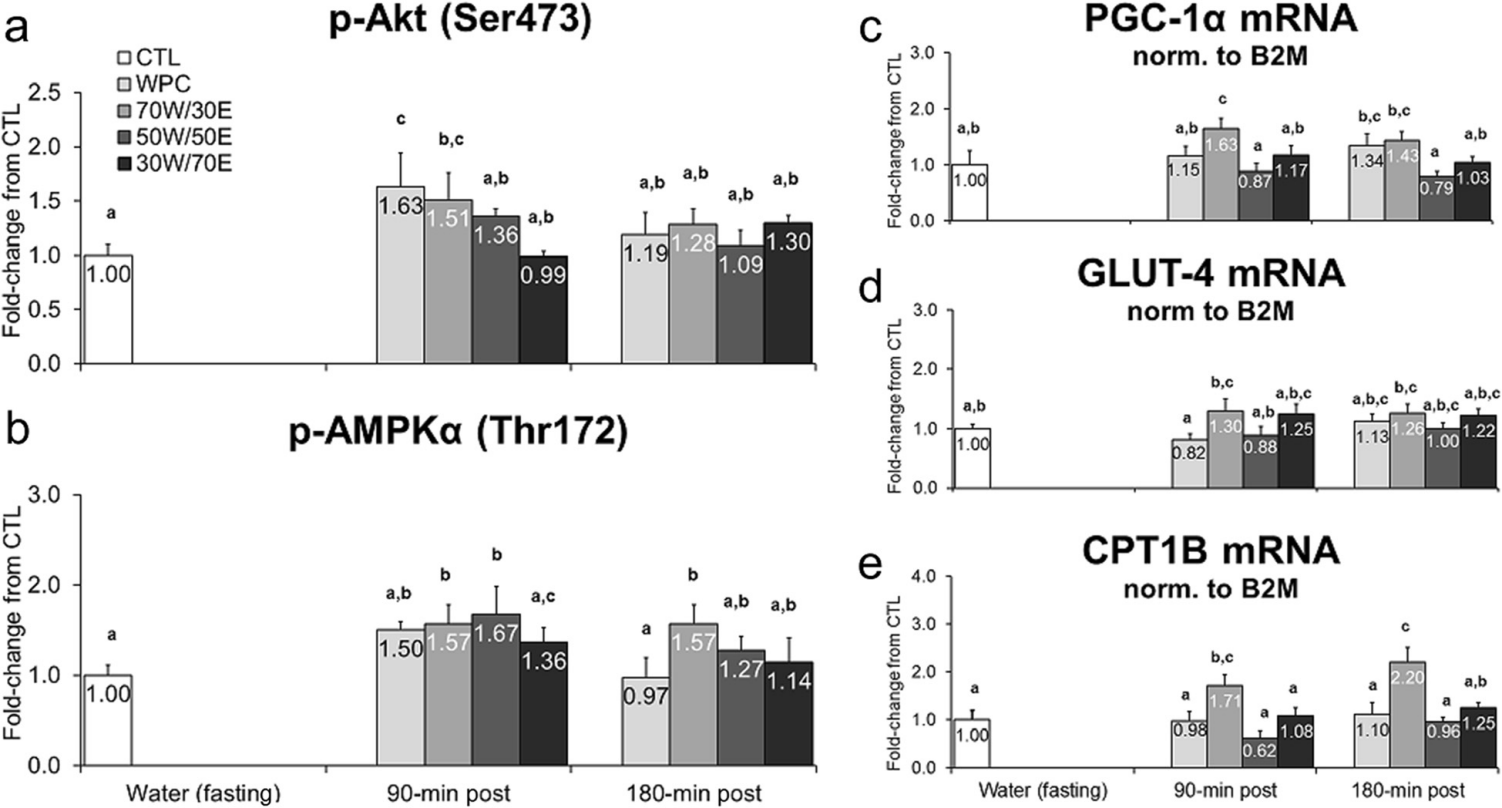
**Effects of different proteins on post-treatment expression of skeletal muscle metabolic markers.** Legend: Effects of each protein on gastrocnemius p-Akt (Ser473) (**panel a**), gastrocnemius p-AMPKα (Thr172) (**panel b**), gastrocnemius PGC-1α mRNA (**panel c**), gastrocnemius GLUT-4 mRNA (**panel d**), and gastrocnemius CPT1B mRNA (**panel e**). Data are presented as means ± standard error (CTL n = 12 per bar, protein groups n = 6–8 per bar). One-way ANOVAs with a Fisher’s LSD *post hoc* test were performed and significant between-treatment differences are represented with different superscript letters (p < 0.05).

While markers of metabolic-related signaling and gene expression in response to feeding provide limited information, these markers also provide putative index regarding whether or not a particular protein source may have a potential impact on long-term metabolic alterations within skeletal muscle. At 90 min post-feeding, WPC and 70 W/30E increased p-Akt (Ser473) compared to CTL rats (WPC vs. CTL p = 0.012, 70 W/30E vs. CTL p = 0.031; Figure 3a), though this increase returned to CTL levels 180 min post-feeding. At 90 min post-treatment, 70 W/30E and 50 W/50E increased p-AMPKα (Thr172) versus CTL rats (70 W/30E vs. CTL p = 0.033, 50 W/50E vs. CTL p = 0.013; Figure 3b), and at 180 min post-treatment 70 W/30E induced a persistent elevation in p-AMPKα (Thr172) versus CTL rats (p = 0.040; Figure 3b). 70 W/30E increased PGC-1α mRNA versus CTL rats 90 min post-treatment (p = 0.002; Figure 3c) and all other protein groups at 90 min post-treatment (70 W/30E vs. WPC p = 0.038, 70 W/30E vs. 50 W/50E p = 0.001, 70 W/30E vs. 30 W/70E p = 0.039; Figure 3c). Though statistical differences existed between treatments for GLUT-4 mRNA, fold-changes between groups were modest (~30%) and there were no clear treatment effects (Figure 3d). Finally, 70 W/30E caused a 1.7-to-2.2-fold increase in CPT1B mRNA versus CTL rats 90 min (p = 0.012; Figure 3e) and 180 min post-treatment (p < 0.001; Figure 3e) as well as other protein groups at 90 min (70 W/30E vs. WPC p = 0.025, 70 W/30E vs. 50 W/50E p = 0.001, 70 W/30E vs. 30 W/70E p = 0.047; Figure 3e) and 180 min post-treatment (70 W/30E vs. WPC p = 0.001, 70 W/30E vs. 50 W/50E p < 0.001, 70 W/30E vs. 30 W/70E p = 0.005; Figure 3e).

### Select lipolysis markers are differentially affected by protein type

**Figure 4:**
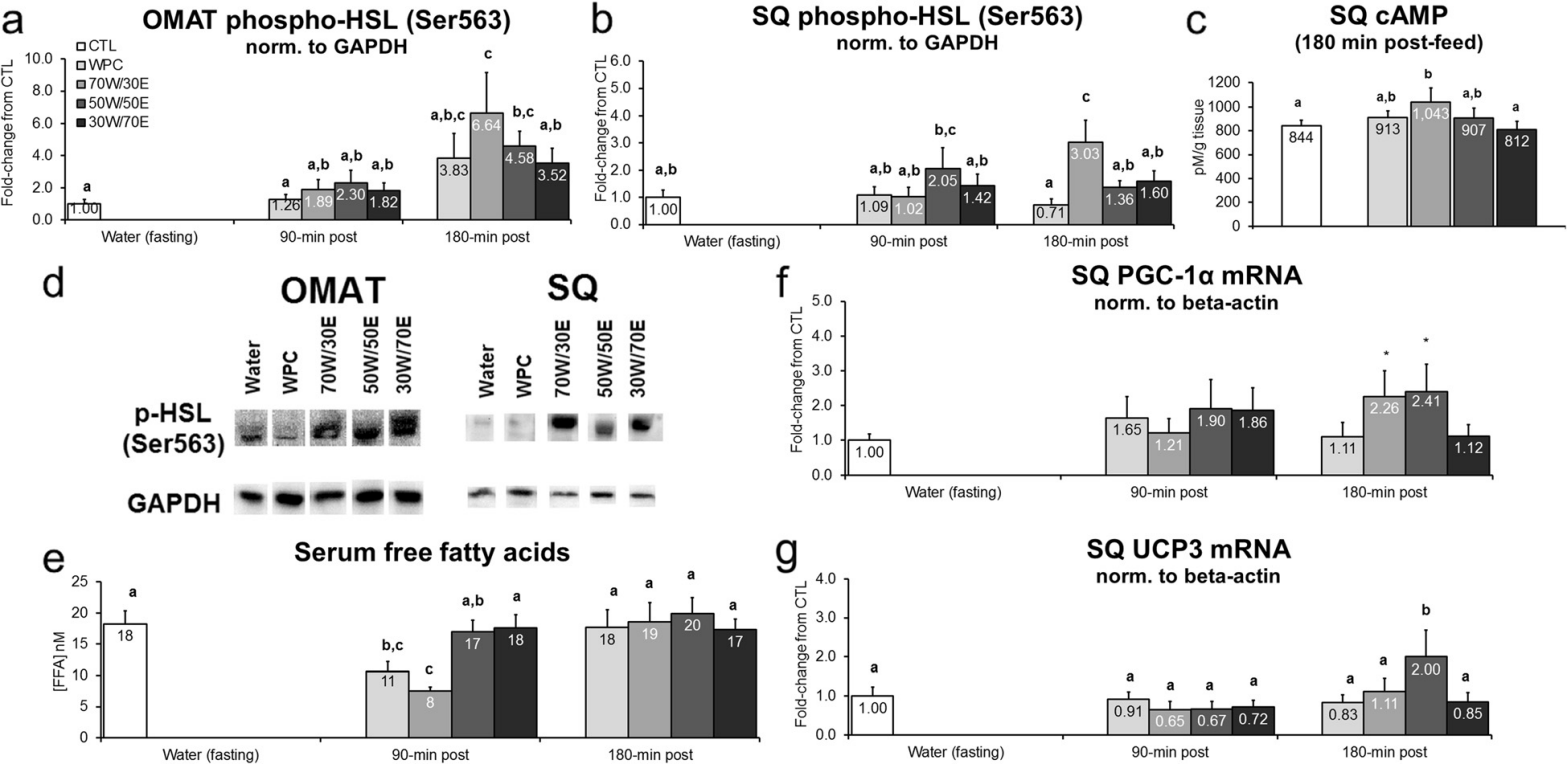
**Effects of different proteins on post-treatment lipolysis markers.** Legend: Effects of each protein on omental adipose tissue (OMAT) p-HSL (Ser563) (**panel a**), subcutaneous adipose tissue (SQ) p-HSL (Ser563) (**panel b**), SQ cAMP tissue concentrations (**panel c**), serum free fatty acid concentrations (**panel e**), SQ PGC-1α mRNA (**panel f**), and SQ UCP3 mRNA (**panel g**). Data are presented as means ± standard error (CTL n = 12–14 per bar, protein groups n = 6–8 per bar). One-way ANOVAs with a Fisher’s LSD *post hoc* test were performed and significant between-feeding differences are represented with different superscript letters (p < 0.05). **Panel d**: representative Western blotting images 180 min post-treatment in OMAT and SQ tissues.

Transient alterations in adipose tissue p-HSL and lipolytic/thermogenic gene expression patterns may provide insight into longer-term alterations that occur at the tissue level (i.e., decrements in fat mass size). Protein feeding did not alter OMAT p-HSL (Ser563) 90 min post-treatment, though WPC, 70 W/30E and 50 W/50E significantly increased this marker 3.8 and 6.5-fold, respectively, 180 min post-feeding versus CTL rats (70 W/30E vs. CTL p < 0.001, 50 W/50E vs. CTL p = 0.019; Figure 4a). Likewise, protein feeding did not alter SQ p-HSL (Ser563) 90 min post-treatment, though 70 W/30 W increased SQ p-HSL (Ser563) 3.1-fold versus CTL rats (p = 0.001; Figure 4b) and 1.9-to-4.4-fold versus all other protein groups 180 min post-treatment (70 W/30E vs. WPC p = 0.001, 70 W/30E vs. 50 W/50E p = 0.015, 70 W/30E vs. 30 W/70E p = 0.035; Figure 3e). Interestingly, 70 W/30E increased SQ cAMP 180 min post-treatment versus CTL rats (p = 0.045; Figure 4c) as well as the 30 W/70E group (p = 0.047; Figure 4c) suggesting that a high proportion of WPH in the test protein may facilitate cAMP-mediated p-HSL activation to increase lipolysis. WPC and 70 W/30E depressed serum free fatty acids 90 min post-treatment versus CTL rats (WPC vs. CTL p = 0.012, 70 W/30E vs CTL p < 0.001; Figure 4e),but this was normalized by 180 min post-treatment. Finally, with regards to thermogenic SQ gene expression markers, 70 W/30E and 50 W/50E tended increase PGC-1α mRNA versus CTL rats 180 min post-treatment (70 W/30E vs. CTL p = 0.083, 50 W/50E vs. CTL p = 0.054; Figure 4f). Furthermore, 50 W/50E increased SQ UCP3 mRNA versus all other protein groups CTL rats 180 min post-treatment (p = 0.004–0.042; Figure 4 g).

### Serum lipolysis and thermogenic hormones are minimally affected by protein type

**Figure 5:**
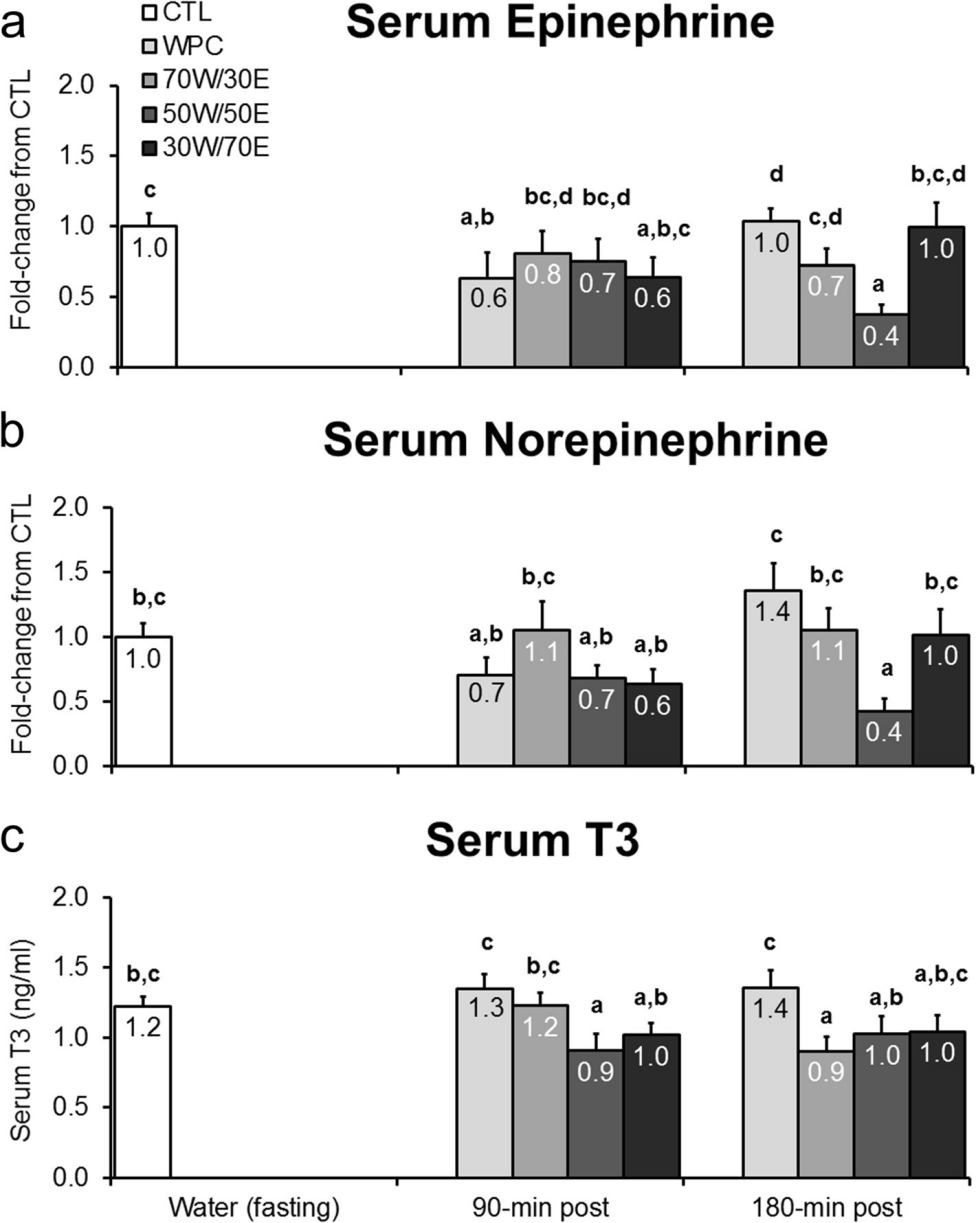
**Effects of different proteins on post-treatment lipolytic/thermogenic hormone markers.** Legend: Effects of each protein on serum epinephrine (**panel a**), norepinephrine (**panel b**), and triiodothyronine (T3) concentrations (**panel c**). Data are presented as means ± standard error (CTL n = 12–14 per bar, protein groups n = 6–8 per bar). One-way ANOVAs with a Fisher’s LSD *post hoc* test were performed and significant between-treatment differences are represented with different superscript letters (p < 0.05).

Given that various OMAT and SQ markers of lipolysis and thermogenesis were differentially affected by different protein types, we next examined if protein feedings affected select hormone levels related to these physiological processes. There was no consistent protein feeding effect on serum catecholamines. WPC and 30 W/70E exhibited 40% lower EPI levels compared to CTL rats 90 min post-treatment (WPC vs. CTL p = 0.039, 30 W/70E vs. CTL p = 0.037; Figure 5a), and 50 W/50E exhibited 60% lower EPI levels compared to CTL rats 180 min post-treatment (p = 0.001; Figure 5a). 50 W/50E exhibited 60% lower NorEPI values compared to compared to CTL rats 180 min post-treatment (p = 0.006; Figure 5b)

There was also no consistent protein feeding effect on serum T3 levels. WPC generally presented greater serum T3 levels versus other treatments 90 and 180 min post-feeding, though these values were not statistically different from fasting rats (Figure 5c). Moreover, 50 W/50E-fed rats exhibited depressed T3 levels compared to CTL rats 90 min post-feeding (p = 0.020; Figure 5c), though this effect was normalized by 180 min post-feeding. Similarly, 70 W/30E-fed rats presented significantly depressed T3 levels by 180 min post-feeding compared to CTL rats (p = 0.023; Figure 5c).

### Effects of different protein feedings on hypothalamic mRNA expression patterns

**Figure 6:**
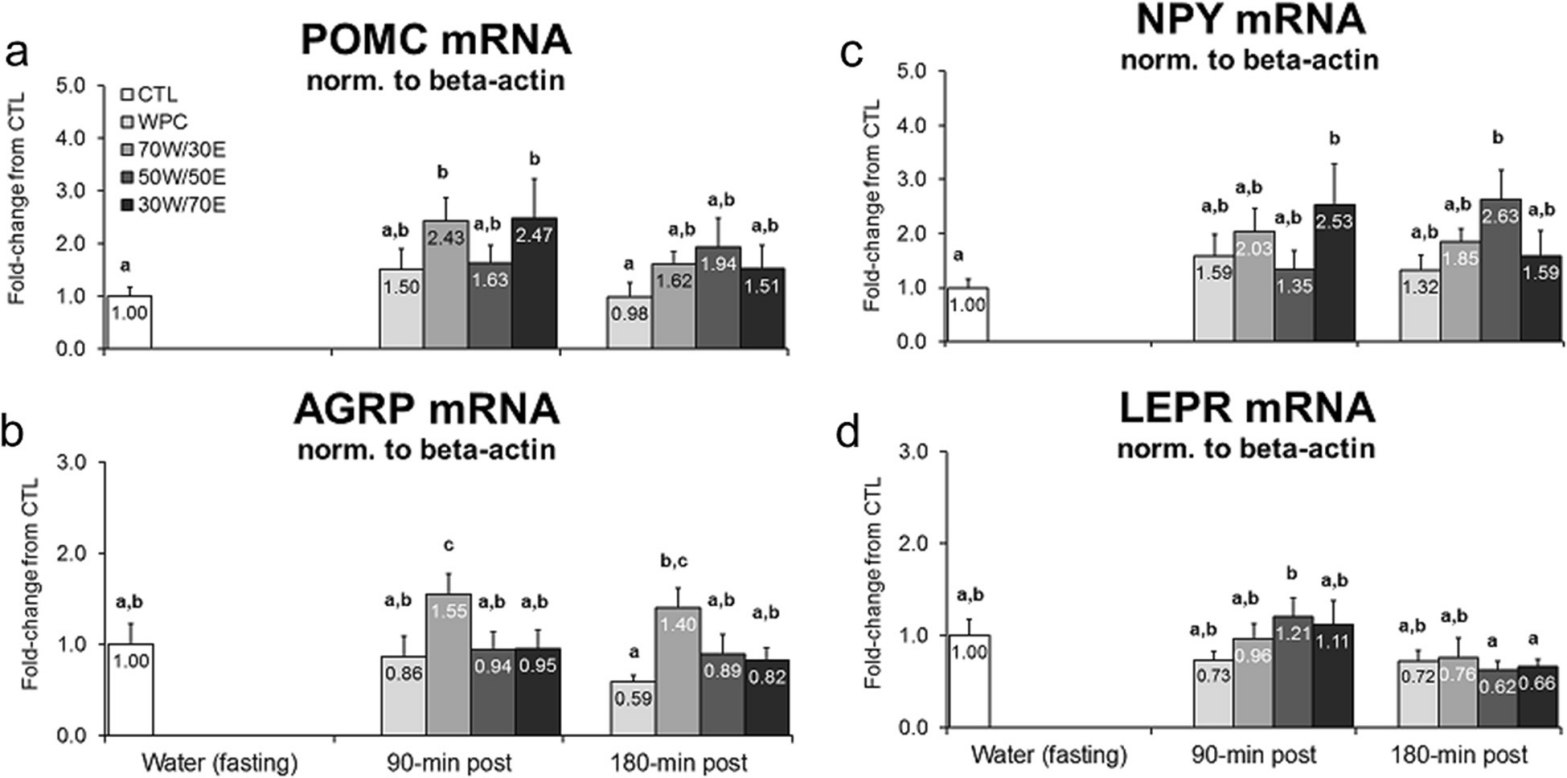
**Effects of different proteins on post-treatment mRNA expression of satiety-related genes.** Legend: Effects of each protein on hypothalamic proopiomelanocortin (POMC) mRNA (**panel a**), agouti-related peptide (AGRP) mRNA (**panel b**), neuropeptide Y (NPY) mRNA (**panel c**), and leptin receptor (LEPR) mRNA (**panel d**). Data are presented as means ± standard error (CTL n = 13 per bar, protein groups n = 5–8 per bar). One-way ANOVAs with a Fisher’s LSD *post hoc* test were performed and significant between-feeding differences are represented with different superscript letters (p < 0.05).

Transient alterations in anorectic and orexigenic gene expression patterns could suggest that an altered satiety response occurs to different protein types. Interestingly, 70 W/30E and 30 W/70E increased hypothalamic POMC mRNA 90 min post-treatment versus CTL rats suggesting a satiety-related response may have occurred in the former groups (70 W/30E vs. CTL p = 0.008, 30 W/70E vs. CTL p = 0.007; Figure 6a). However, there was a compensatory increase in orexigenic AGRP mRNA in the 70 W/30E group 90 min post-treatment versus CTL rats (p = 0.040; Figure 6b). Likewise, there was a compensatory increase in orexigenic NPY mRNA in the 30 W/70E group 90 min post-treatment versus CTL rats (p = 0.032; Figure 6c), and a significant increase in this marker in the 50 W/50E group 180 min post-treatment versus CTL rats (p = 0.009; Figure 6c). Though statistical differences existed between groups for hypothalamic LEPR mRNA, fold-changes between protein groups and CTL rats were modest and non-significant (±20–40%, p > 0.05; Figure 6d).

## Discussion

### Protein type is an important factor in acutely increasing markers of skeletal muscle anabolism

Whey and egg protein consumption has been posited to promote anabolic effects in skeletal muscle via greater post-feeding increases in serum amino acids versus other protein sources [2]. All test proteins in the current study increased the phosphorylation status of mTOR, p70s6k, and rps6 90 min post-feeding compared to CTL rats, though 70 W/30E-fed rats presented sustained elevations in phosphorylated mTOR and rps6 180 min post-feeding. These phosphorylated targets are positive effectors of MPS, and our findings are in agreement with past literature suggesting that whey and egg protein increase the phosphorylation of one or more of these intramuscular signaling markers following feeding with [19,34,20,35] or without [2,1] resistance exercise in rats and humans. However, it is intriguing that higher proportions of EPH (i.e., 50–70%) did not statistically increase MPS levels versus CTL rats. Norton et al. [1] demonstrated that a test meal containing 0.64 g of whey or egg protein similarly increases MPS 90 min post-feeding. Our study differs from the findings of Norton et al. given that: a) MPS was measured using two different methodologies; specifically we used the SUnSET method and Norton et al. used an L-^2^ H_5_-phenylalanine tracer; b) Norton et al. measured post-feeding MPS at 90 min while we measured MPS 180 min post-feeding; and c) Norton et al. fed rats 0.64 g protein in a solid mixed-meal form while we fed rats 0.19 g of unadulterated test protein solutions. In spite of these methodological differences, we suggest that, relative to CTL rats, a low protein dose comprised mainly of whey protein (i.e., WPC or 70 W/30E) promotes a greater post-feeding increase in MPS relative to a low dose protein solution comprised primarily of egg protein. Alternatively stated, while egg protein is a source of leucine and EAAs, it appears that whey protein is superior at stimulating MPS at lower doses in the current rodent model. While this seems contrary to the conclusions posited by Norton et al. suggesting that the high leucine content in whey and egg equally stimulate MPS, two independent human studies have demonstrated that younger [36] and older subjects [37] consuming supplemental egg protein while resistance training do not experience increases in muscle mass after 8–12-week interventions. Specifically, Hida et al. [36] demonstrated that 15 g/d of egg protein supplementation in female athletes, who were engaged in a resistance training protocol, increased lean body mass by 1.5 kg, whereas a carbohydrate placebo increased lean body mass by 1.6 kg. Likewise, Iglay et al. [37] demonstrated that supplementing the diet with an additional 20 g/d of egg protein did not further increase the lean mass or skeletal muscle cross-sectional area compared to a lower protein group when both groups resistance trained for 12 weeks; of note, both groups gained roughly 1.0 kg of lean body mass.

In contrast, a recent meta-analysis examining several studies [5] clearly demonstrates that whey protein supplementation with resistance exercise is effective at increasing muscle mass in younger and older populations, and Phillips et al. [6] noted that participants engaged in 8–16 weeks of resistance exercise gain, on average, 3.0 kg of lean mass compared to 1.0 kg of lean mass gains in the placebo groups of these studies. One hypothesis deserving of future investigation is whether mammary-derived proteins, due to the inherent purpose of such proteins promoting rapid growth and development of offspring, may offer unique physiological advantages versus what can otherwise be labeled as ‘nutritional protein sources’ such as egg or other animal proteins. In this regard, future studies examining why a low dose of whey protein is unique in stimulating muscle anabolism relative to other protein sources that possess a ‘leucine-, BCAA-, and EAA-rich profile’ are warranted.

### Putative anabolic and atrogene gastrocnemius mRNA responses following different protein feedings

Akirin-1/Mighty increased approximately 90% 180 min post-feeding in the WPC and 70 W/30E groups versus CTL rats and other protein groups. Akirin-1/Mighty is a transcriptional target of MSTN that is related to controlling myotube size *in vitro* [32], and resistance exercise has been shown to transiently up-regulate Akirin-1/Mighty mRNA in rodent skeletal muscle [31]. To our knowledge, only one other recent study to date has determined that certain akirin genes are transcriptionally up-regulated in fish that were fasted 21 days and then re-fed [38]. Hence, the aforementioned study along with our current data suggests that Akirin-1/Mighty mRNA is sensitive to protein feeding, and this finding should be further examined at the mechanistic level in order to determine if whey protein affects skeletal muscle hypertrophy through increases in Akirin-1/Mighty mRNA expression.

The expression of select anabolic and catabolic-related gastrocnemius mRNAs responded differently between different treatment groups. Interestingly, higher proportions of EPH caused 90–180 min increases in MSTN mRNA versus CTL rats and/or higher proportions of whey protein. Preliminary data in humans suggest that the consumption of fertile egg yolk powder reduces circulating MSTN levels [25]. Hence, if one or multiple putative bioactive components in egg protein extract reduce serum MSTN levels then it is possible that skeletal muscle may undergo a compensatory increase in skeletal MSTN mRNA expression to counter systemic down-regulation. Thus, while our data and other limited evidence suggests that MSTN expression is responsive to dietary egg proteins, more research is needed in order to elucidate if egg protein-induced increases in MSTN gene expression and/or signaling in skeletal muscle results in a physiological meaningful response.

All protein sources generally increased the p21Cip1 mRNA expression 180 min post-feeding compared to CTL rats suggesting that protein feeding in general regulates the expression of this gene. p21Cip1 gene expression has been theorized to promote satellite cell differentiation [39,40], though limited information suggests that p21Cip1 gene expression up-regulates protein synthesis and pathological hypertrophy in kidney epithelial cells [41]. Thus, it will be of further interest to examine if protein feeding-induced increases in skeletal muscle p21Cip1 gene expression are related to post-mitotic skeletal muscle protein synthesis mechanisms.

Atrogin-1 was up-regulated in 70 W/30E-fed rats 180 min post-feeding versus CTL rats. Similarly, MuRF-1 was up-regulated in WPC-fed and 70 W/30E-fed rats 180 min post-feeding versus CTL rats. Our finding that test solutions containing predominantly whey protein increase postprandial atrogene (atrogin-1 and MuRF-1) mRNA expression is intriguing given that amino acids are thought to be anti-catabolic [42]. However, ingesting smaller protein ingestion boluses (10–20 g) have been reported to increase MuRF-1 mRNA in human skeletal muscle after resistance exercise versus a larger bolus (40 g) [43]. Thus, our finding that protein ingestion increases the mRNA expression of select atrogenes may represent a stimulation of greater muscle protein turnover rather than an increase in atrophic mechanisms.

### Protein source and type as important factors in acutely affecting markers of skeletal muscle metabolism and reduced muscle catabolism

Higher proportions of whey protein in the test solutions (i.e., WPC and 70 W/30E) increased Akt phosphorylation (Ser473) 90 min post-feeding versus CTL rats. Tissue Akt phosphorylation at the Ser473 residues is a common readout for insulin signaling and sensitivity [44], and whey protein feeding following resistance exercise in humans has been shown to increase Akt phosphorylation at the Ser473 residue [19,20]. Our findings are also in partial agreement with West et al. [45] who demonstrated in humans that an EAA bolus increases skeletal muscle Akt phosphorylation (Ser473) 60 min after feeding. As noted above, however, WPC and EPH are also a rich source of EAAs. Thus, we speculate that the increase in Akt phosphorylation in the WPC and 70 W/30E groups may have been due to the superior ability of whey protein in stimulating insulin secretion and, thus, downstream insulin signaling in skeletal muscle. While we did not measure serum insulin responses in the current study, we have previously shown that WPH feeding to rats causes a robust (>2-fold) rise in insulin 60 min post-feeding [23]. Hence, foods containing a higher proportion of whey protein may stimulate greater intramuscular insulin signaling, and future research should continue to examine if WPC or WPH feeding in acute and long-term settings can enhance insulin sensitivity in insulin-resistant subjects.

Interestingly, 70 W/30E feeding caused a 63% increase in skeletal muscle PGC-1α mRNA expression versus CTL rats, as well as a significant increase in this gene relative to all other groups 90 min post-treatment. Furthermore, rats fed 70 W/30E exhibited a significant increase in skeletal muscle CPT1B mRNA 90- and 180 min post-feeding; this being a gene which is involved with fatty acid transport to the mitochondria for fuel oxidation. Whey protein isolate has been shown to stimulate a further increase in PGC-1α mRNA expression in human skeletal muscle 6 h following cycling [46]. However, to our knowledge, this is the first study to demonstrate that a test protein containing chiefly WPH can increase post-feeding skeletal muscle PGC-1α mRNA expression independent of exercise. We posit that one potential mechanism whereby WPH stimulates the mRNA expression of PGC-1α and CPT1B is through the stimulation of AMPK activity (Figure 3a). To this end, Canto et al. [47] have demonstrated that AMPK activation increases the expression of these two genes, and this would support the hypothesis that whey protein, in particular WPH, can stimulate oxidative metabolism and mitochondrial biogenesis with long-term supplementation. This hypothesis is not unfounded given recent evidence that prolonged whey protein feeding has been shown to increase mitochondrial content and respiration in the brain [48] and liver [49]. Therefore, more mechanistic studies should examine if WPH administration increases the post-feeding expression of mitochondrial-related genes via AMPK activation and/or other mechanisms.

### Effects of different proteins on post-feeding markers of lipolysis

As mentioned prior, whey protein ingestion exerts positive effects on body composition and fat mass [14,5]. Furthermore, and as mentioned previously, WPH supplementation during exercise may provide added benefit to reducing body fat versus intact/native protein sources. Despite a transient 90 min post-feeding depression in serum FFAs with 70 W/30E feeding versus CTL rats and other protein groups, 50–70% WPH protein feedings increased select markers of adipose tissue lipolysis and thermogenesis 180 min post-feeding. For instance, rats fed 70 W/30E presented increases in SQ cAMP levels as well as OMAT and SQ p-HSL (Ser563). Likewise, rats that were fed higher proportions of WPH (e.g., 70 W/30E or 50 W/50E) exhibited increases in SQ PGC-1α and UCP3 mRNA expression levels which are putative markers of adipose tissue thermogenesis [50]. Finally, 70 W/30E increased gastrocnemius CPT1B mRNA which could be suggestive of a potential long-term enhancement in fatty acid transport to the mitochondria for oxidation. Conversely, circulating catecholamine levels in response to feeding higher proportions of WPH exhibited no discernable effects. These findings are difficult to reconcile as we have previously reported that WPH increases serum EPI 30 min post-feeding versus WPC-fed and CTL rats [18]. Therefore, the 180-min post-feeding increase in lipolysis markers in the current study may be due to an earlier increase in catecholamines (i.e., within 60 min of feeding) which was not captured due to sampling time points and/or due to WPH-borne bioactives that selectively act upon adipose tissue to stimulate lipolytic mechanisms.

Of note, we measured serum T3 given that it is a well-known stimulator of thermogenesis and cellular respiration. With regards to adipose tissue lipolysis, T3 has been shown to increase adipocyte beta-adrenergic receptor which, in turn, increases lipolytic capabilities over longer-term periods [51]. Notwithstanding, there was no clear protein feeding effect on serum T3 depression, and T3 values did not seem to parallel the increased lipolysis and thermogenesis markers in rats fed 70 W/30E or 50 W/50E which refutes the potential role of thyroid hormones in facilitating this effect.

One final mechanistic explanation as to how higher proportions of WPH increased lipolysis markers is through potential tricarboxylic acid (TCA) cycle modulation. To this end, a recent study by Lillefosse et al. [52] demonstrated that chronic whey protein feeding to obese-prone rodents significantly reduced fat mass gain in response to concomitant high fat feeding. The authors suggested that whey protein feeding increases the urinary excretion of TCA substrates which are stimulators of fatty acid synthesis [53]. Alternatively stated, the ability of WPH to ‘extract’ TCA cycle intermediates from adipose tissue during the post-feeding period may place adipose tissue in a catabolic state thereby initiating lipolysis-related mechanisms. This is not unfounded, as we have previously noted that WPH significantly increases circulating TCA intermediates (i.e., citrate, succinate, fumarate and malate) 60 min post-feeding versus WPC-fed rats (*supplementary data* in [18]). Hence, more research is needed regarding if the depletion of TCA cycle intermediates within adipose tissue is linked to the WPH-induced lipolysis response.

### Effects of different proteins on post-feeding markers of satiety

Sousa et al. [54] recently posited that, regardless of protein source, amino acids may reduce appetite via an increase in gut hormone secretion, an increase in anorexigenic POMC gene expression in the hypothalamus, and/or a reduction in orexigenic NPY gene expression in the hypothalamus. 70 W/30E and 30 W/70E increased hypothalamic POMC mRNA expression patterns 90 min post-feeding; this being a marker that favors satiety signaling in the hypothalamus [55]. However, there was a compensatory increase in the orexigenic AGRP transcript in rats fed a high proportion of WPH. Furthermore, some protein feedings induced an increased expression of hypothalamic NPY mRNA versus CTL rats which, again, suggests a potential orexigenic versus satiety response. Therefore, our mixed findings suggest that two possibilities may exist including: a) the amount of total protein fed to rats, while beneficial in stimulating skeletal muscle anabolism and adipose tissue lipolysis, was not entirely effective at initiating a satiety response; and/or b) hypothalamic signaling is so tightly regulated that a post-feeding increase in anorectic genes is countered with a compensatory increase in orexigenic genes.

Finally, it should be noted that the post-feeding effects of each protein on hypothalamic LEPR mRNA expression patterns was of considerable interest due to the central role of leptin receptor signaling in satiety. Thus, we initially hypothesized that protein-feeding induced alterations in LEPR mRNA expression may be a potential culprit in initiating longer-term body composition alterations through enhanced satiety mechanisms that have been reported to previously occur with chronic protein supplementation. To this end, McAllan et al. [11] recently performed a long-term rodent feeding study whereby C57BL/6 J mice were fed a high fat diet (HFD, 45% energy as fat) enriched with either 20% energy as casein or whey protein isolate. HFD feeding increased the hypothalamic mRNA expression of LEPR; an effect which the authors suggest may be a hallmark feature of hyperphagia and obesity development. However, mice that were co-fed whey protein isolate with the HFD presented a significant reduction in hypothalamic LEPR mRNA expression. Notwithstanding, we demonstrated no noticeable between-group differences in LEPR mRNA expression patterns which suggests that the hypothalamic expression gene is not appreciably altered after one feeding and/or LEPR gene expression may be indiscriminately regulated more so by amino acid concentration alone as opposed to specific bioactive peptides.

## Conclusions

We have demonstrated that protein type provide uniquely different physiological responses over a transient post-prandial time course. Specifically, and seemingly irrespective of protein type, administering higher concentrations of whey versus egg protein to healthy rodents causes: a) a greater anabolic response in rodents with regards to post-feeding MPS compared to a fasting condition; and b) an increase in intramuscular insulin sensitivity markers (i.e., Akt signaling markers and transient increases in PGC-1α mRNA expression patterns). Alternatively, the administration of higher concentrations of WPH versus EPH increases select markers of post-feeding lipolysis 3 h post-feeding. Of note, while we make assertions that whey protein forms may be more beneficial in facilitating increases in muscle mass and fat loss compared to egg protein per the current findings, the acute nature of this study is a pervading limitation of these hypotheses. Likewise, while several of tissue markers were statistically altered in response to different protein feedings, more research is needed comparing whey versus egg protein supplementation on longer-term physiologically-relevant outcomes (i.e., increases in muscle mass, decreases in fat mass, and/or alterations in satiety as suggested by our transient findings reported herein). Therefore, further research is this nutraceutical arena is warranted with regards to how protein source and type (i.e., native versus hydrolyzed), and varying combinations thereof may affect these physiological parameters in over more chronic periods and in more clinical-based populations.

## Additional file

Additional file 1: Figure S1 Preliminary testing different WPC doses on post-feeding gastrocnemius phosphorylated mTOR markers and muscle protein synthesis 90 min post-treatment. Legend: data are presented as means ± standard error [CTL n = 8 per bar except for MPS where n = 3 per bar, WPC groups n = 2–3 per bar]. One-way ANOVAs with a Fisher’s LSD *post hoc* test was performed; * indicates significance versus water (fasting) rats (p < 0.05). These data show that a low dose of WPC (0.19 g which is 10 human eq. g) is just as effective at stimulating most mTOR substrates and MPS levels versus moderate (0.37 g which is 19 human eq. g) and high (0.93 g which is 19 human eq. g) WPC doses. The relatively low dose (0.19 g which is 10 human eq. g) was subsequently employed for WPC, 70 W/30E, 50 W/50E and 30 W/70E comparisons.
