# Supplementary figures and images for: Effects of protein type and composition on postprandial markers of skeletal muscle anabolism, adipose tissue lipolysis, and hypothalamic gene expression
Source: J Int Soc Sports Nutr. 2015 Mar 13;12:14. doi: 10.1186/s12970-015-0076-9 (PMC4365970; doi:10.1186/s12970-015-0076-9)

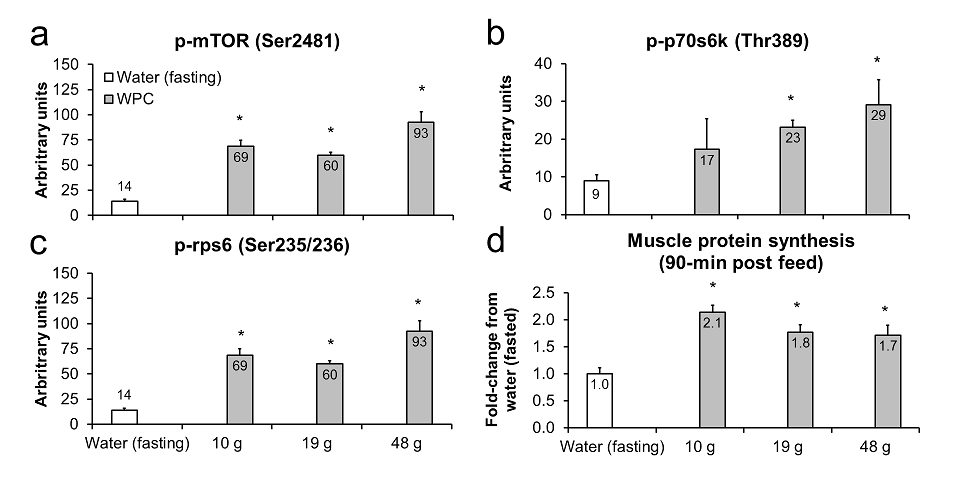

Supplement: Additional file 1: Figure S1 — Preliminary testing different WPC doses on post-feeding gastrocnemius phosphorylated mTOR markers and muscle protein synthesis 90 min post-treatment. Legend: data are presented as means ± standard error [CTL n = 8 per bar except for MPS where n = 3 per bar, WPC groups n = 2–3 per bar]. One-way ANOVAs with a Fisher’s LSD post hoc test was performed; * indicates significance versus water (fasting) rats (p < 0.05). These data show that a low dose of WPC (0.19 g which is 10 human eq. g) is just as effective at stimulating most mTOR substrates and MPS levels versus moderate (0.37 g which is 19 human eq. g) and high (0.93 g which is 19 human eq. g) WPC doses. The relatively low dose (0.19 g which is 10 human eq. g) was subsequently employed for WPC, 70 W/30E, 50 W/50E and 30 W/70E comparisons. [file 12970_2015_76_MOESM1_ESM.png]
